# Upregulation of Cystathionine *β*-Synthetase Expression Contributes to Visceral Hyperalgesia Induced by Heterotypic Intermittent Stress in Rats

**DOI:** 10.1371/journal.pone.0053165

**Published:** 2012-12-28

**Authors:** Yongmeng Wang, Ruobing Qu, Shufen Hu, Ying Xiao, Xinghong Jiang, Guang-Yin Xu

**Affiliations:** Institute of Neuroscience, Key Laboratory of Pain Basic Research and Clinic Therapy, Department of Neurobiology, Soochow University, Suzhou, P. R. China; Zhejiang University School of Medicine, China

## Abstract

**Background:**

Hydrogen sulfide (H_2_S) functions as a neuromodulator, but whether it modulates visceral pain is not well known. This study was designed to determine the role for the endogenous H_2_S producing enzyme cystathionine *β*-synthetase (CBS) and cystathionine *γ*-lyase (CSE) in a validated rat model of visceral hyperalgesia (VH).

**Methods:**

VH was induced by nine-day heterotypic intermittent stress (HIS). Abdominal withdrawal reflex (AWR) scores were determined by measuring the visceromoter responses to colorectal distension (CRD). Dorsal root ganglia (DRG) neurons innervating the colon were labeled by injection of DiI (1,1'-dioleyl-3,3,3',3-tetramethylindocarbocyanine methanesulfonate) into the colon wall. Patch clamp recording techniques were employed to examine excitability and sodium channel currents of colon specific DRG neurons. Tissues from colon related thoracolumbar DRGs were analyzed for CBS, CSE and sodium channel expression.

**Results:**

HIS significantly increased the visceromotor responses to CRD in association with an upregulated expression of CBS not CSE proteins in colon related DRGs. Administration of *O*-(Carboxymethyl)hydroxylamine hemihydrochloride (AOAA), an inhibitor of CBS, attenuated the AWR scores in HIS-treated rats, in a dose dependent fashion. In contrast, AOAA did not produce any effect on AWR scores in healthy control rats. AOAA reversed the potentiation of sodium channel current densities of colon specific DRG neurons of HIS rats. To further confirm the role for CBS-H_2_S signaling, NaHS was used to mimic the production of H_2_S by CBS. Application of NaHS significantly enhanced neuronal excitability and potentiated sodium channel current densities of colon DRG neurons from healthy control rats. Furthermore, AOAA reversed the upregulation of Na_V_1.7 and Na_V_1.8 in colon related DRGs of HIS rats.

**Conclusion:**

Our results suggest that upregulation of CBS expression might play an important role in developing VH via sensitization of sodium channels in peripheral nociceptors, thus identifying a specific neurobiological target for the treatment of VH in functional bowel syndromes.

## Introduction

Irritable bowel syndrome (IBS), a common functional gastrointestinal disorder, is characterized by altered bowel evacuation, bloating and visceral pain in the absence of anatomical or biochemical abnormalities [1], [2]. Up to date, the etiologies of these symptoms are still not fully understood. Recent studies have showed that stress is related with symptom onset, exacerbation and perpetuation in patients with functional gastrointestinal disorders [3]. Early life stressors, like childhood neglect, parental influence, physical or social abuse, life threatening situations, have been shown to increase the risk of IBS development [4], [5], [6]. Clinical studies show that chronic stress plays an important role in the pathophysiology of IBS [7]. Altered visceral sensitivity with increased responses to colorectal distension (CRD) consistently presents and is recognized as a hallmark of IBS in clinic [8], [9], [10]. Therefore, stress-induced visceral hypersensitivity is proposed to be a significant component in the pathophysiology of IBS. However, the precise mechanism of stress-induced visceral hypersensitivity remains unknown.

H_2_S is a malodorous and poisonous gas, acting as an endogenous neuromodulator/gasotransmitter as well as an intracellular messenger, like nitric oxide and carbon monoxide. It is generated from L-cystenine by two pyridoxal-5-phosphate (PLP)-dependent enzymes, cystathionine *β*-synythase (CBS) and cystathionine *γ*-lyase (CSE) in the mammalian body [11], [12] or 3-mercaptopyruvate sulfurtransferase (3MST), the PLP-independent enzyme mainly in the brain [13]. Recent studies have shown that H_2_S involves in many physiological and pathophysiological function and processes at molecular, cellular, tissue, and system levels [14], [15], [16]. In the colon, H_2_S was reported to regulate smooth muscle tone [17] and function as a novel nociceptive messenger in the guinea-pig, rats and humans under normal and inflammatory conditions [18], [19], [20]. However, whether H_2_S plays a role under chronic stress-induced visceral pain remains elusive. Mechanisms for the above described biological actions of H_2_S include activation of ATP-sensitive K^+^ channels [21], [22], [23], mitogen-activated protein kinase pathways [24], or T-type Ca^2+^ channels [18], [25] or exciting the capsaicin-sensitive sensory neurons [19], [26]. We have previously reported that H_2_S donor NaHS significantly enhanced the frequency of action potentials of colon specific DRG neurons [14], but the ionic mechanism is not clear. Membrane ion channels including voltage-gated sodium and potassium channels play a fundamental role in controlling neuronal excitability. Both the upregulation of sodium currents and/or suppression of potassium currents appear to contribute to peripheral sensitization [27], [28]. In the present study, we hypothesized that heterotypic intermittent stress (HIS) elevated CBS gene expression and led to an increase in H_2_S formation, which sensitizes voltage-gated sodium channels, thus contributing to HIS-induced visceral hyperalgesia (VH). To test this hypothesis, we investigated roles of HIS and H_2_S on neuronal excitability, sodium channels function and expression. We found that CBS-H_2_S signaling was involved in HIS-induced visceral hyperalgesia. HIS led to a significant upregulation of CBS expression which was associated with an enhanced AWR scores and sodium channel activities. Inhibition of CBS attenuated AWR scores and suppressed sodium current density. H_2_S donor enhanced the neuronal excitability and increased sodium current density as well. Our findings suggest that CBS-H_2_S signaling is crucial for stress-induced VH.

## Materials and Methods

### Animals

Adult male Sprague-Dawley rats, weighing 250±20 g, were obtained from the Experimental Animal Center of Soochow University. Animals were housed under controlled conditions (07∶00∼19∶00 lighting, 24±2°C) with free access to a standard laboratory diet and fresh water. Care and handling of these animals were approved by the Institutional Animal Care and Use Committee of the Soochow University and were in accordance with the guidelines of the International Association for the Study of Pain.

### Heterotypic Intermittent Stress Protocol

Rats were subjected to 9 consecutive days of a heterotypic intermittent stress (HIS) protocol composed of 3 randomly arranged stressors, 60 minutes of water avoidance stress, 45 minutes of cold restraint stress at 4°C, or 20 minutes of forced swimming stress, as described previously [29].

### Measurement of Visceromoter Response to Graded CRD

Visceral hypersensitivity was measured by grading the response of rats to colorectal distention (CRD) as described previously [30], [31], [32]. Briefly, rats were lightly sedated with diethyl ether while a flexible balloon (6 cm) made of a surgical glove finger attached to a tygon tubing was inserted 8 cm into the descending colon and rectum via the anus and held in place by taping the tubing to the tail. Rats were placed in small Lucite cubicles and allowed to adapt for 30 minutes. CRD was performed by rapidly inflating the balloon to a constant pressure measured using a sphygmomanometer connected to a pressure transducer. The balloon was inflated to various pressures: 20, 40, 60 and 80 mmHg, for a 20 seconds stimulation period followed by a 2 min rest. Behavioral responses to CRD were measured by visual observation of the abdominal withdrawal reflex (AWR) by a blinded observer and the assignments of an AWR score were as follows: 0 = Normal behavior without response; 1 = Brief head movement at the onset of the stimulus followed by immobility; 2 = Contraction of abdominal muscles; 3 = Lifting of the abdomen off the platform; 4 = Body arching and lifting of pelvic structures. In addition, colonic distension threshold, the minimal pressure to induce abdominal muscle contraction, was also used to measure the time course of HIS and drug effects.

### Western Blotting

Protein extracts from bilateral TL (T13-L2) DRGs of control and HIS-treated rats were prepared in MT-CelLytics mammalian tissue protein extraction reagent, 1 mM PIC, 1∶100 dilution of protease inhibitor cocktail (Biocolor BioScience & Technology Company, CHN). Twenty micrograms (20 µg) of proteins were fractionated on 10% polyacrylamide gels (Bio-Rad). Proteins were transferred to polyvinyldifluoride (PVDF) membranes (Roche) at 200 mA for 2 hours at 4°C. Membranes were blocked for 2 hours in TBS (50 mM Tris-Base, 133 mM NaCl, pH = 7.4) and 5% dilution of non-fat milk powder. Primary antibody (anti-CBS and anti-CSE at 1∶1000, Abnova, Taiwan CHN; rabbit anti-Na_V_1.7 and anti-Na_V_1.8 at 1∶200, Alomone, Israel) was incubated for 2 hours in 1% milk TBS at room temperature. After washing in TBST (0.5% Tween-20), membranes were incubated with HRP conjugated secondary antibodies (1∶4000, MultiSciences Biotech Co., CHN) in TBS and 1% milk for 2 hours at room temperature. Bands were visualized using ECL (Biological Industries, CHN) and exposed to Kodak X-ray film. Membranes were subsequently stripped and re-probed for β-actin (1∶1000, MultiSciences Biotech Co., CHN). Films were scanned and band intensities were measured using Optic Quant software (ImageJ, NIH). CBS, CSE, Na_V_1.7 and Na_V_1.8 data were expressed normalized to β-actin.

### Whole-cell Patch Clamp Recordings

As described previously [31], DRGs (T13-L2) were dissected out and incubated in dissecting solution with enzymes (collagenase D, 1.5–1.8 mg/ml, Roche and trypsin, 1.2 mg/ml, Sigma) for 1.5 hour at 34.5°C. DRGs were then taken from the enzyme solution, washed, and transferred to 2 ml of the dissecting solution containing DNase (0.5 mg/ml, Sigma). Single cell suspension was subsequently obtained by repeat trituration through flame-polished glass pipettes. Coverslips containing adherent DRG cells were put in a small recording chamber (1 ml volume) and attached to the stage of an inverted microscope (Olympus IX71, Japan) fitted for both fluorescence and bright-field microscopy. DiI-labeled neurons were identified by their fluorescence under the fluorescent microscope. Single cell activities were sampled at 100 µs per point and filtered at 2–5 KHz. For patch-clamp recording experiments, normal external solution contained (in mM): 130 NaCl, 5 KCl, 2 KH_2_PO4, 2.5 CaCl_2_, 1 MgCl_2_, 10 HEPES, 10 glucose (pH = 7.2–7.3 adjusted with NaOH; osmolarity = 295–300 mOsm). Recording pipettes were pulled from borosilicate glass tubing using a horizontal puller (P-97, Sutter Instruments) and typically had a resistance of 3–5 MΩ when filled with normal pipette solution containing (in mM): 140 KCl, 10 NaCl, 5 EGTA, 1 CaCl_2_, 10 HEPES, 10 glucose (pH = 7.25 adjusted with KOH; osmolarity = 306 mOsm). For patch-clamp recordings of sodium currents, pipette solution contained (in mM) 140 CsF, 1 MgCl_2_, 5 EGTA, 3 Na-GTP, 10 HEPES, 10 glucose (pH = 7.2 adjusted with CsOH; osmolarity = 285–295 mOsm), while bath solution contained (in mM) 60 NaCl, 80 choline chloride, 0.1 CaCl_2_, 10 TEA-Cl, 10 HEPES, 10 glucose, 0.1 CdCl_2_ (pH = 7.4 adjusted with TEA-OH; osmolarity = 310 mOsm). Action potential and voltage-gated sodium currents were recorded with whole-cell patch clamp techniques by a patch-clamp amplifier (HEKA Elektronik, Lambrecht, GER) and stored for offline analysis.

### Drug Application

For behavioral experiments, *O*-(Carboxymethyl)hydroxylamine hemihydrochloride (AOAA) or normal saline (NS) was intraperitoneally (i.p.) injected 5.5 hours after the last stressor. Thirty minutes after injection, behavioral tests were performed. This procedure ensured that the behavioral studies were carried out 6 hours after the last stressor. For whole-cell patch clamp experiments or protein measurements, AOAA or NS was i.p. administrated 30 minutes before stressor starting from the sixth day to the ninth day during the HIS protocol, and once more 24 hours after the last stressor. Thus, AOAA was injected for 5 consecutive days. This procedure was obtained by our pilot study that neuronal excitability was reduced by at least 5 times of AOAA injection. Animals were euthanized 30 min after injection and the colon related DRGs were harvested for patch clamp recordings or gene expression analyses.

### Data Analysis

All data are expressed as mean ± SEM. Statistical analysis were conducted using commercial software OriginPro 8 (OriginLab, US) and Matlab (Mathworks, US). Normality was checked for all data before analyses. Significance was determined using paired sample *t*-Test, two sample *t*-Test, paired sample sign test, Mann-Whitney test, Kruskal-Wallis ANOVA, Dunn’s post hoc test following Friedman ANOVA, one-way ANOVA, one-way repeated-measures ANOVA or two-way repeated-measures ANOVA followed by Tukey post hoc test, as appropriate. The level of significance was set at *p*<0.05.

## Results

### HIS-induced Visceral Hypersensitivity was Associated with an Upregulation of CBS Expression

**Figure 1:**
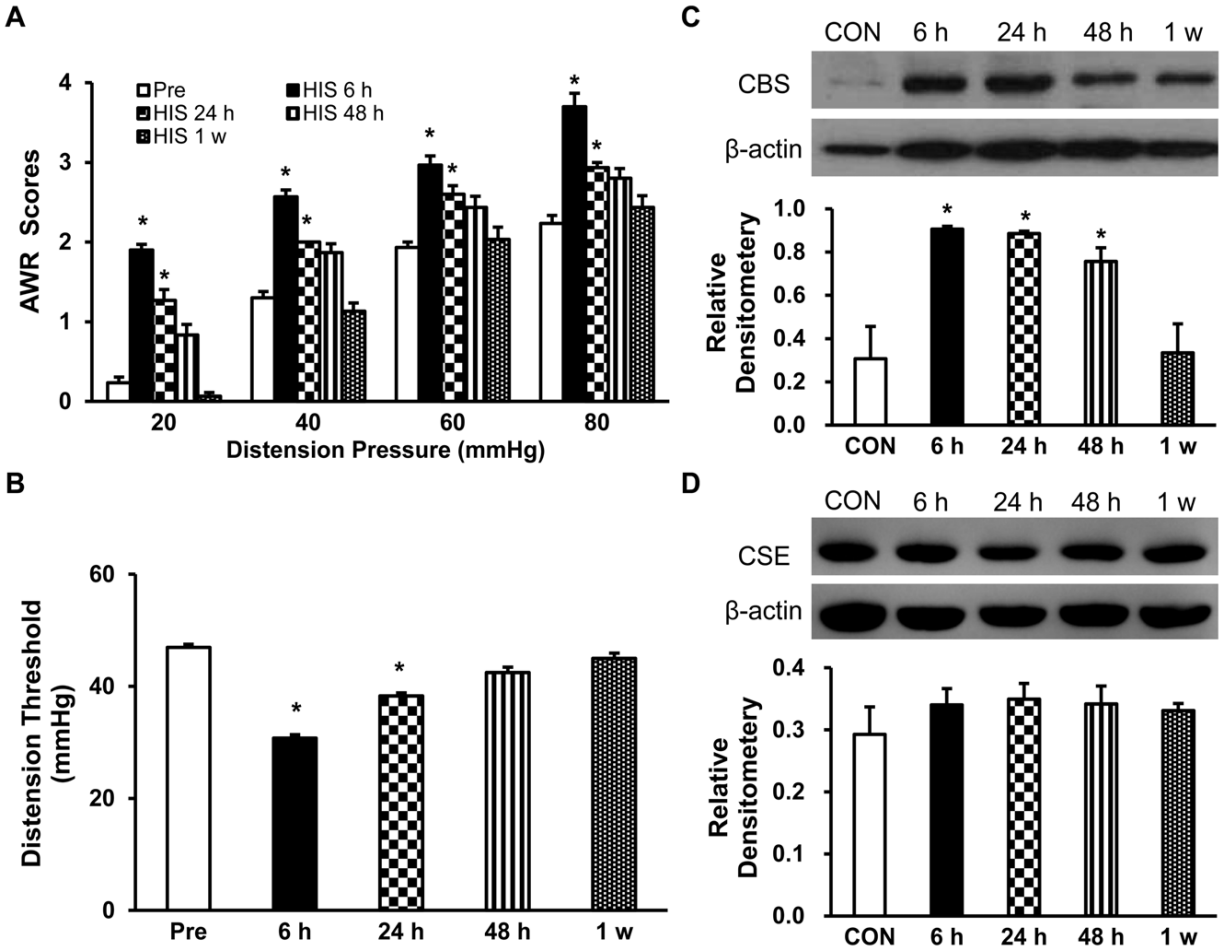
Visceromotor response to CRD and CBS expression following HIS. (A) HIS significantly increased AWR scores in rats responding to CRD compared with that of pre-stress baseline (Pre). AWR scores started to increase at 6 h and returned to normal level 48 hours after HIS. n = 10 rats; *p<0.05 vs. Pre. (B) The distention threshold was also reduced 6 and 24 h after termination of the last stressor and returned to pre-stress level 48 h after termination of the last stressor. n = 10 rats, *p<0.05 vs. Pre. (C) Representative image of western blots showing the time course of CBS expression in colon related DRGs (T13-L2) following HIS. Actin control for each sample was given. Expression levels of CBS was greatly elevated 6 hours after HIS and remained at high level for 48 hours, and returned to control levels by 1 week. n = 3–4 rats for each time points; *p<0.05 vs. control (CON). (D) Representative image of western blots showing the time course of CSE expression in colon related DRGs (T13-L2) following HIS. HIS treatment did not significantly alter the expression of CSE in colon related DRGs.

In agreement with our previous report [29], heterotypic intermittent stress (HIS) significantly increased the visceromotor responses to graded CRD at pressures of 20, 40, 60 and 80 mmHg at 6 h and 24 h after HIS, by measuring AWR scores (Fig. 1A, n = 10 rats for each group; *p<0.05 vs. Pre group, Dunn`s post hoc test following Friedman’s ANOVA) and distention threshold (Fig. 1B, n = 10 rats for each group; *p<0.05 vs. Pre group, Dunn`s post hoc test following Friedman’s ANOVA) compared to pre-stressed baseline (Pre). To determine whether HIS induced upregulation of CBS expression, bilateral thoracolumbar (T13, L1, L2) DRGs were dissected out 6, 24, 48 hours or 1 week after termination of the last stressor. As shown in figure 1C, HIS significantly increased CBS expression in T13-L2 DRGs at 6, 24 and 48 h and the expression was returned to normal level 1 week after termination of the last stressor (Fig. 1C, HIS 6 h/CON = 2.95; HIS 24 h/CON = 2.88; HIS 48 h/CON = 2.46; HIS 1 w/CON = 1.09; n = 3 rats for control and 1 week; n = 4 rats for 6, 24 and 48 h; *p<0.05 vs. CON, one-way ANOVA followed by Tukey post hoc test). In contrast to CBS expression, CSE expression was not altered at any time point after HIS treatment (Fig. 1D, n = 3 rats for each group).

### CBS Inhibitor Reduced Visceromotor Responses in HIS Rats

**Figure 2:**
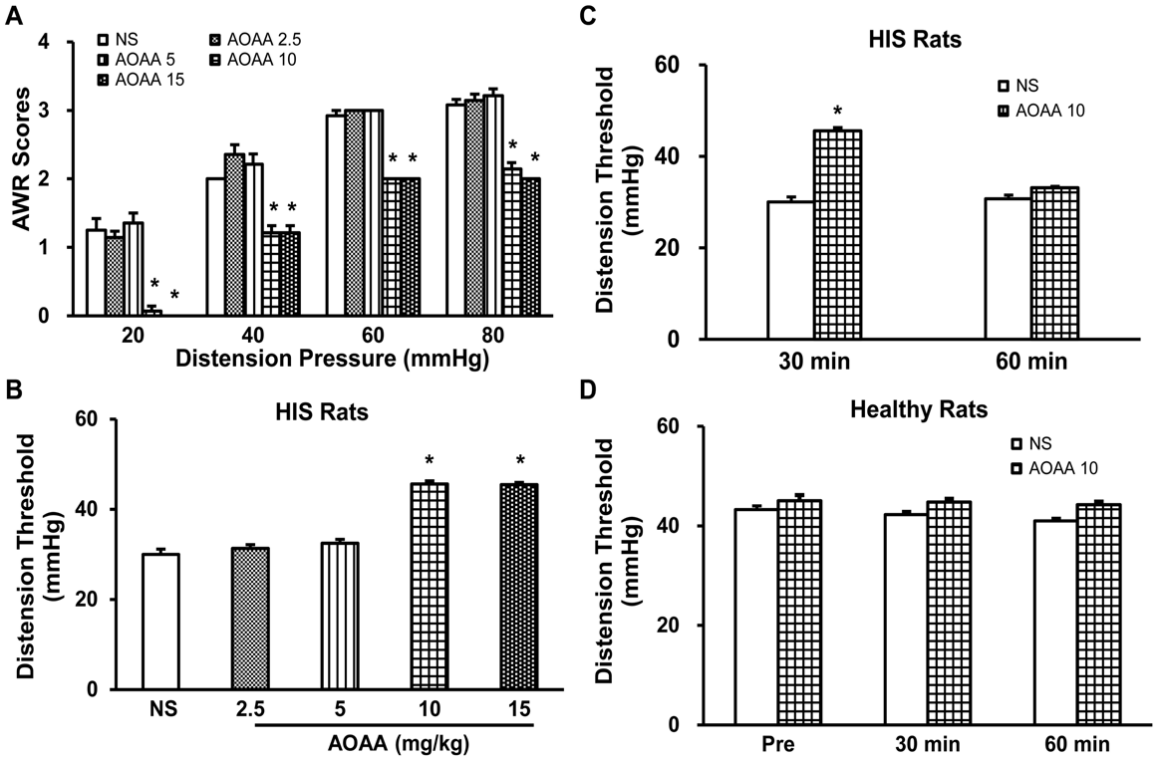
The reversal of HIS-induced visceral hyperalgesia by pretreatment with CBS inhibitor AOAA. (A) AOAA at 10–15 mg/kg attenuated the HIS-induced increase in AWR scores. *p<0.05 vs. NS, n = 7 rats for each group. (B) AOAA also increased distension threshold. *p<0.05 vs. NS, n = 7 rats for each group. (C) The time course of distension threshold 60 min after AOAA (10 mg/kg, i.p.). *p<0.05 vs. NS, n = 7 rats for each group. (D) NS or AOAA did not produce any effect on distension threshold in control healthy rats, n = 5 for each group.

To determine whether H_2_S is involved in HIS-induced visceral hypersensitivity, the H_2_S-producing enzyme CBS inhibitor, AOAA, was administrated. Since CSE expression was not altered, further investigation of CSE role was not included in this study. Intraperitoneal (i.p.) injection of AOAA had a significant effect on the AWR scores in HIS rats (Fig. 2, Friedman ANOVA). The administration of AOAA 30 minutes before CRD attenuated the AWR scores in HIS rats in a dose dependent manner (Fig. 2A, n = 7 rats for each group; *p<0.05 vs. NS, Tukey post hoc test following Kruskal-Wallis ANOVA). To further confirm the effect of systemic administration of AOAA, distention threshold, the minimal pressure to evoke abdominal visceromotor responses, was determined. Administration of AOAA also enhanced distention threshold, in a dose-dependent manner (Fig. 2B, n = 7 rats for each group, *p<0.05 vs. NS, Tukey post hoc test following Kruskal-Wallis ANOVA). The optimized dose for AOAA to produce the maximal effect was 10 mg/kg body weight in this study. We then determined the time course of AOAA effects. The effect of AOAA at 10 mg/kg lasted ∼30 min (Fig. 2C, n = 7 rats for each group; *p<0.05 vs. NS, Tukey post hoc test following two-way repeated-measures ANOVA). AOAA at 10 mg/kg or NS had no significant effects on the distension threshold in healthy control rats (Fig. 2D, n = 5 rats for each group), suggesting that this agent did not act as a non-specific analgesic and that CBS do not normally participate in the responses to CRD in normal conditions.

### CBS Inhibitor Reversed the Potentiation of Current Densities of VGSCs of Colon-specific DRG Neurons

**Figure 3:**
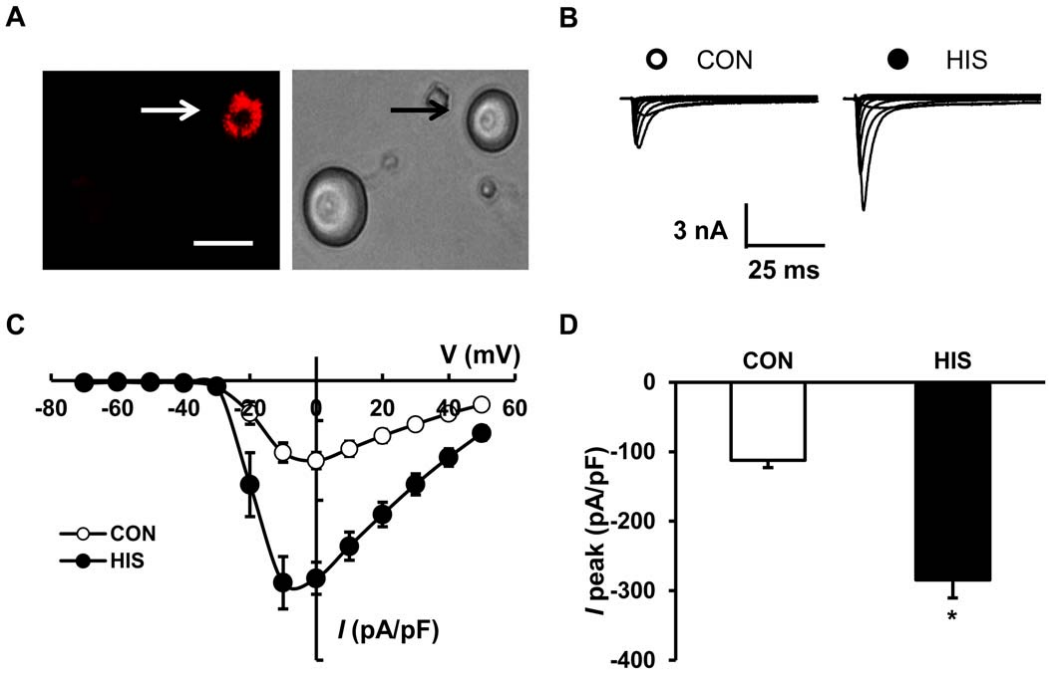
Potentiation of voltage-gated sodium currents in DiI-labeled neurons from HIS rats. (A) An example of a Dil-labeled T13-L2 DRG neuron (Left). A phase image of the same neuron is shown on the right. Bar = 35 µm. For recording of voltage-gated sodium currents, the membrane potential was held at −60 mV and voltage steps were from −70 mV to +50 mV with 10 mV increments and 80 ms duration. (B) Examples of voltage-gated sodium currents recorded from control (left) and HIS rats (right). (C) *I*–V curves for sodium currents of control (CON) and HIS-treated rats (n = 17 neurons for each group). Each point represented means ± SEM. (D) HIS treatment significantly increased the peak amplitude of sodium currents compared with age-matched control rats (n = 17 neurons for each group), *p<0.05.

The previous data showed that HIS-induced visceral hypersensitivity is associated with an increase in excitability of colon-specific TL DRG neurons [29]. We examined the ionic mechanism underlying the enhanced neuronal excitability in this study. Voltage-gated sodium channels (VGSCs) are responsible for the generation and propagation of action potentials in the membranes of neurons. Colon-specific DRG neurons were labeled by DiI (Fig. 3A). Using the whole-cell patch clamp technique, we first measured current densities of VGSCs in DiI-labeled DRG neurons from control and HIS rats (Fig. 3B). The current-voltage relationship was also examined (Fig. 3C). The average reversal potentials were 70.01±2.81 mV (n = 17 neurons) and 66.57±2.10 mV (n = 17 neurons) for control and HIS rats, respectively. HIS treatment did not significantly alter the reversal potential of sodium currents (p>0.05), indicating that ion permeability was not changed. However, HIS significantly increased the average of peak amplitude of sodium currents in DiI-labeled neurons compared with the control (CON: −113.90±10.32 pA/pF; HIS: −284.44±25.86 pA/pF; n = 17 neurons for each group, *p<0.05, two sample *t*-Test; Fig. 3D). These results support our previous hypothesis that HIS-induced hyperexcitability is mediated, at least in a large part, by sensitization of sodium channel currents in colon-specific DRG neurons [29].

**Figure 4:**
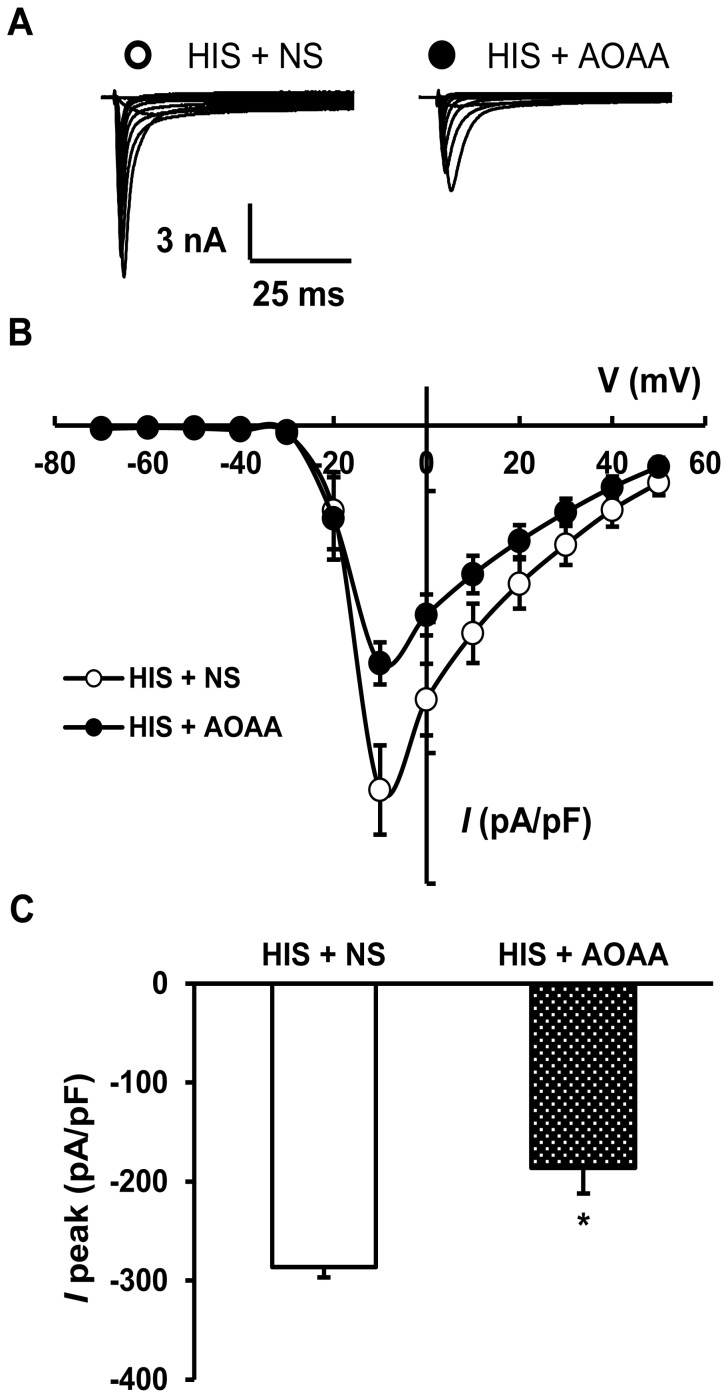
Inhibitory effect of AOAA on the potentiation of voltage-gated sodium currents. (A) Examples of voltage-gated sodium currents recorded from normal saline (left) and AOAA-treated rats (right). (B) *I*–V curves for sodium currents of colon-specific DRG neurons recorded from HIS rats treated with NS or AOAA (n = 12 neurons for each group). Each point represented means ± SEM. (C) AOAA treatment reversed the potentiation of peak amplitude of sodium currents compared with NS-treated rats (n = 12 neurons for each group); *p<0.05.

Since AOAA attenuated the AWR scores in HIS rats, we next investigated whether AOAA suppressed current density of VGSCs in colon-specific DRG neurons (Fig. 4). Rats were divided into two groups: AOAA group treated with AOAA (10 mg/kg, i.p.) and NS group treated with the same volume of normal saline (NS, i.p.). Colon specific DRG neurons were harvested 24 hours after termination of the last stressor. AOAA treatment significantly reduced the current density of VGSCs compared with the NS-treated group (HIS+NS: −286.45±33.43 pA/pF; HIS+AOAA: −188.01±18.27 pA/pF, n = 12 neurons for each group, *p<0.05, Mann-Whitney test; Fig. 4B and C). However, AOAA treatment did not significantly change the reversal potentials. The average reversal potentials were 65.37±2.03 mV (n = 12 neurons) and 61.74±1.82 mV (n = 12 neurons) for NS and AOAA, respectively (Fig. 4B, *p*>0.05), indicating that ion permeability was not changed after AOAA treatment.

### The H_2_S Donor NaHS Increased Current Densities of VGSCs and Neuronal Excitability of Colon-specific DRG Neurons

**Figure 5:**
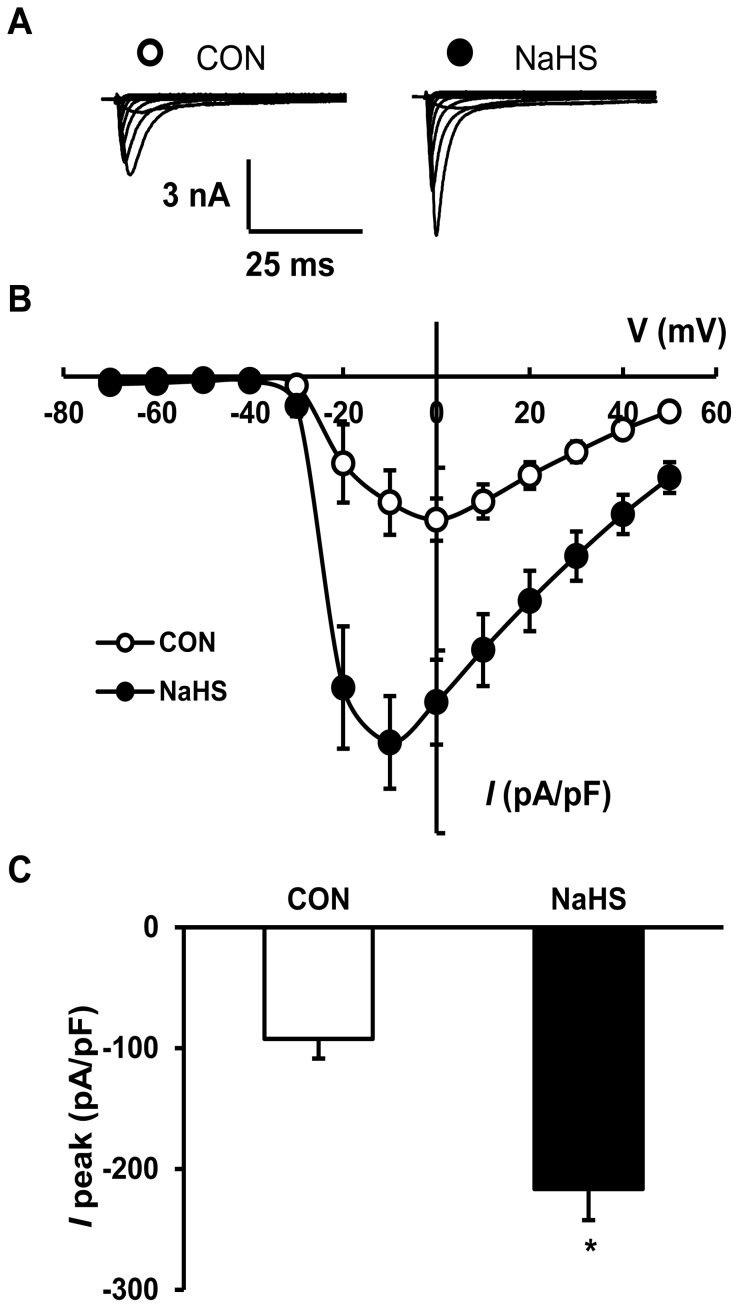
Potentiation of sodium channel current densities by NaHS application. (A) Examples of voltage-gated sodium currents recorded from colon specific DRG neurons treated with control (left) and 3 min after perfusion of NaHS (250 µM, right). (B) *I*–V curves for sodium currents recoded from colon specific DRG neurons treated with control (CON, n = 11 neurons) and NaHS (n = 13 neurons). (C) NaHS application remarkably increased the peak amplitude of sodium currents compared with controls (CON, n = 11 neurons; NaHS, n = 13 neurons, *p<0.05).

To further confirm the role for H_2_S, NaHS, a donor of H_2_S, was used in this study. Addition of NaHS mimics the role of CBS to produce H_2_S. NaHS was freshly prepared in the concentration of 250 µM and added into the recording chamber for 3 min. External solution for recording sodium currents was used as control. NaHS application significantly increased peak current density of VGSCs compared with controls (Fig. 5C, CON: −92.29±16.23 pA/pF, n = 11 neurons; NaHS: −216.47±25.97 pA/pF, n = 13 neurons; *p<0.05, two sample *t*-Test). However, NaHS treatment did not significantly change the reversal potentials. The average reversal potentials were 64.38±2.22 mV (n = 11 neurons) and 70.43±2.27 mV (n = 13 neurons) for CON and NaHS, respectively (Fig. 5B, *p*>0.05).

**Figure 6:**
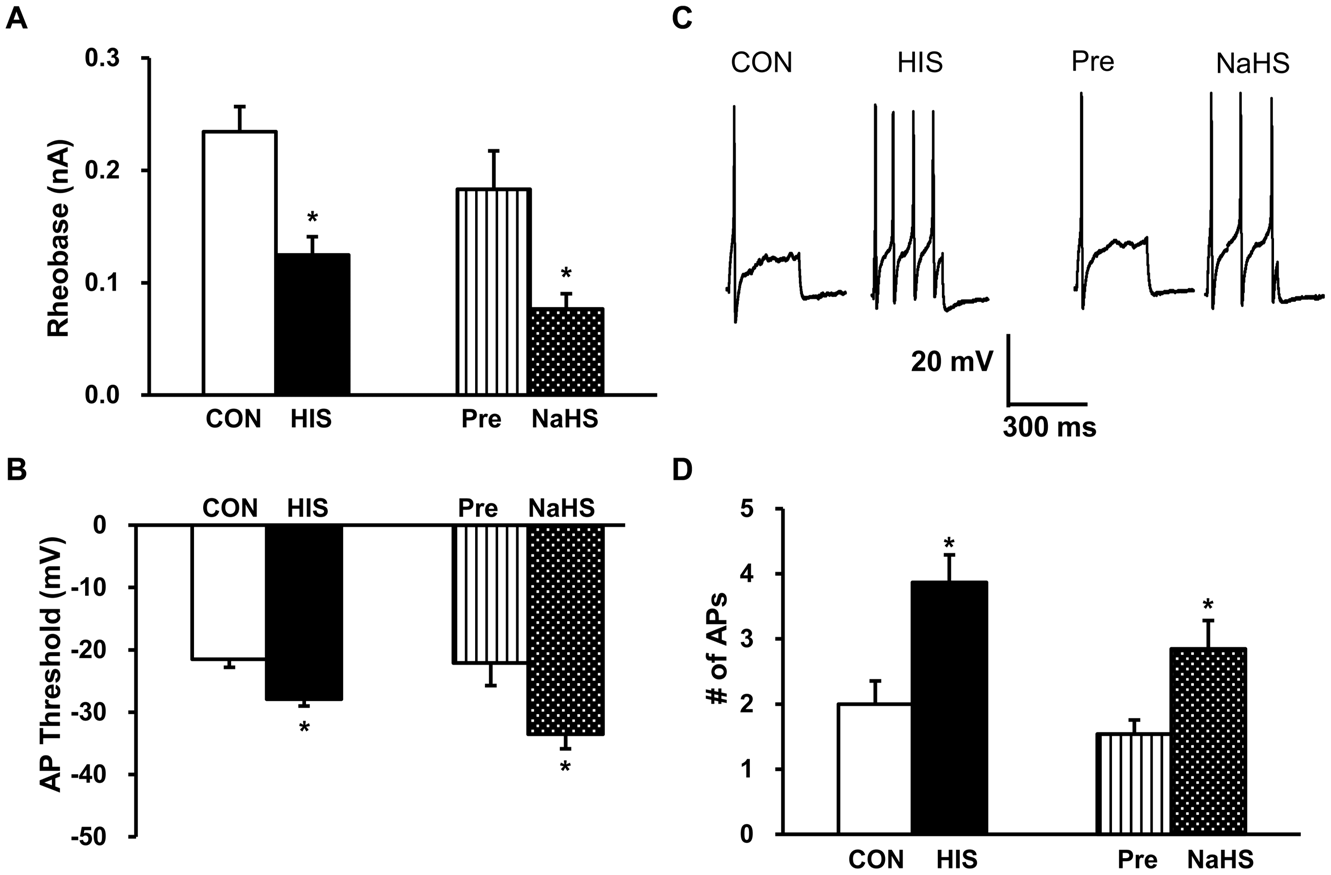
Increase in excitability of colon-specific DRG neurons from control healthy rats by NaHS application. (A) Bar graph showing decreases in rheobase in HIS rats and in NaHS-treated group when compared with their respective controls (CON, n = 18 neurons; HIS, n = 15 neurons; Pre and NaHS, n = 13 neurons; *p<0.05). (B) Graph showing hyperpolarization of AP threshold in HIS rats and in NaHS-treated group when compared with their respective controls (CON, n = 18 neurons; HIS, n = 15 neurons; Pre and NaHS, n = 13 neurons; *p<0.05). (C) Representative traces of APs were induced by 300 ms depolarizing current pulses injected through the patch pipette at two times rheobase in DiI labeled neurons from control and HIS rats, and treated without and with NaHS. (D) Bar graph showing a significant increase in average number of APs elicited by a two times rheobase current injection from HIS rats and neurons treated with NaHS. n = 13 neurons, *p<0.05 compared with Pre.

We then determined the role for H_2_S on neuronal excitability. In an agreement with our previous report [29], HIS treatment significantly reduced the rheobase (Fig. 6A, CON: 0.23±0.02, n = 18 neurons; HIS: 0.13±0.02, n = 15 neurons; *p<0.05, two sample *t*-Test), hyperpolarized the AP threshold (Fig. 6B, CON: −21.53±1.28, n = 18 neurons; HIS: −27.94±1.08, n = 15 neurons; *p<0.05, two sample *t*-Test) and increased the number of APs in response to 2 times rheobase current stimulation (Fig. 6C and D, CON: 2.00±0.35, n = 18 neurons; HIS: 3.87±0.42, n = 15 neurons; *p<0.05, Mann-Whitney test). These data further support our previous view that the excitability of colon-specific DRG neurons was enhanced after HIS treatment. We next defined the role for H_2_S on TL DRG neurons isolated from healthy control rats. Incubation of these neurons with freshly prepared NaHS (250 µM) for 3 min significantly decreased the rheobase (Fig. 6A, Pre: 0.18±0.03; Post: 0.08±0.01; n = 13 neurons; *p<0.05, paired sample sign test) and hyperpolarized AP threshold (Fig. 6B, Pre: −22.11±3.63; Post: −33.53±2.34; n = 13 neurons; *p<0.05, paired sample *t*-Test). Application of NaHS also increased the numbers of APs evoked by 2 times rheobase current stimulation in TL DRG neurons from healthy control rats. The average number of APs after NaHS application were significantly higher than those before NaHS application (Fig. 6D, Pre: 1.54±0.22; Post: 2.85±0.44; n = 13 neurons; *p<0.05, paired sample sign test). These data suggested that H_2_S donor NaHS mimics the effect of HIS, thus producing visceral hypersensitivity.

### AOAA Treatment Reversed Upregulation of Na_V_1.7 and Na_V_1.8 Expression

**Figure 7:**
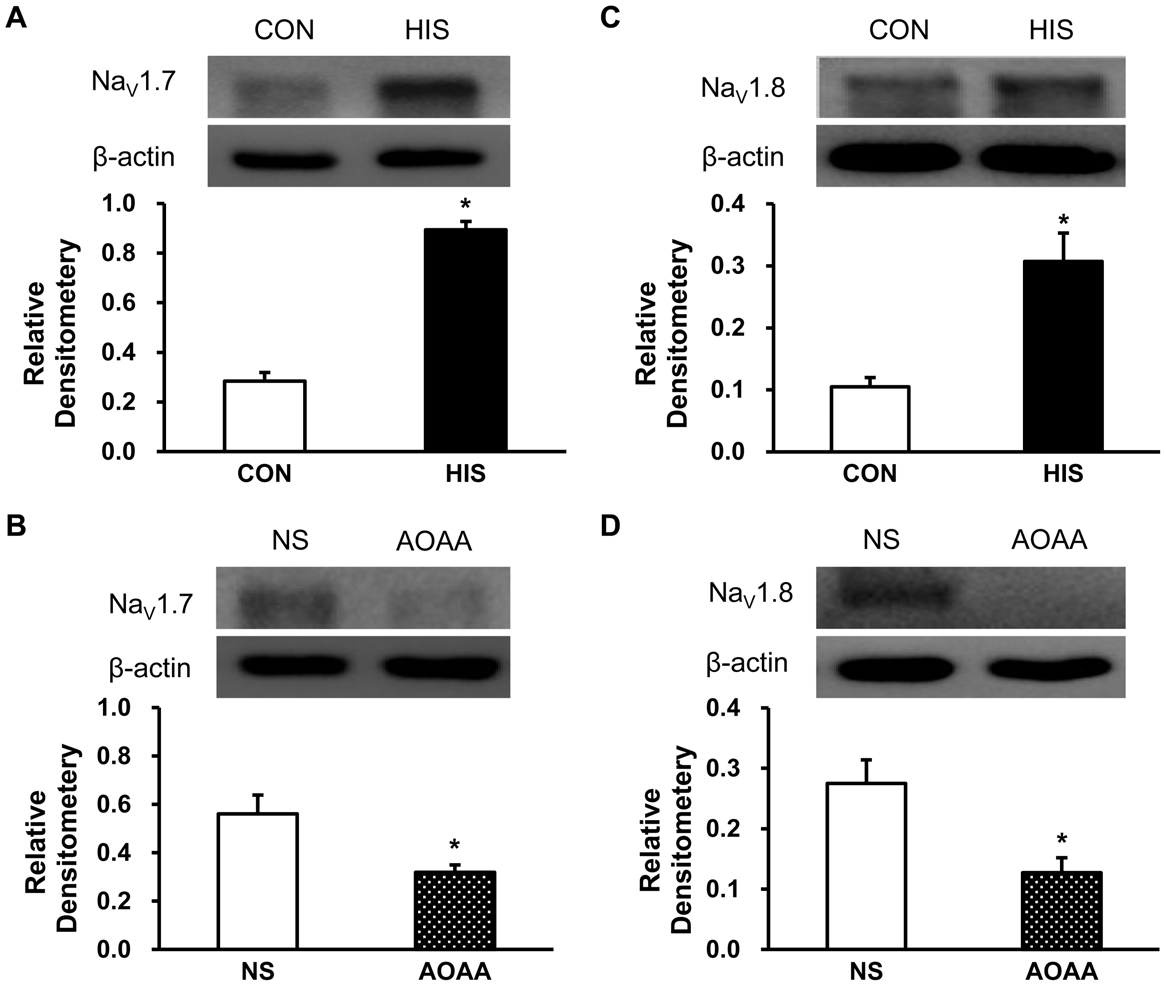
Reversal by AOAA of upregulation of Na_V_1.7 and Na_V_1.8 expression. (A) Western blots for Na_V_1.7 expression from ganglia of control (CON) and HIS rats. Actin control for each sample was given. After HIS, the relative density of Na_V_1.7 was increased by 214% (n = 3 rats; *p<0.05). (B) Western blots for Na_V_1.7 from ganglia of HIS rats treated with normal saline (NS) or AOAA. AOAA treatment significantly reduced Na_V_1.7 expression (n = 3 rats; *p<0.05). (C) Western blots for Na_V_1.8 expression from ganglia of control (CON) and HIS rats. After HIS, the relative density of Na_V_1.8 was increased by 247% (n = 4 rats; *p<0.05). HIS treatment enhanced Na_V_1.7 and Na_V_1.8 expression. (D) Western blots for Na_V_1.8 from ganglia of HIS rats treated with normal saline (NS) or AOAA. AOAA treatment significantly reduced Na_V_1.8 expression (n = 3 rats; *p<0.05).

To determine whether the expression of Na_V_1.7 and Na_V_1.8 indeed increased in DRG after HIS, western blotting assays were performed on colon related DRGs in control and HIS rats. Proteins were isolated from T13-L2 DRGs of control rats and rats treated with HIS 24 hours after termination of the last stressor. After separating the proteins by electrophoresis under denaturing conditions, they were transferred to PVDF membranes and probed with anti-Na_V_1.7 (Fig. 7A) or anti-Na_V_1.8 (Fig. 7C). After HIS treatment, the level of expression of Na_V_1.7 and anti-Na_V_1.8 was increased dramatically (Fig. 7A, HIS/CON = 3.14, n = 3 rats for each group; *p<0.05, two sample *t*-Test. Fig. 7C, HIS/CON = 3.47, n = 4 rats for each group; *p<0.05, two sample *t*-Test). Thus, HIS upregulates both Na_V_1.7 and Na_V_1.8 expression in colon related DRGs. To determine the role for CBS in the upregulation of Na_V_1.7 and Na_V_1.8, AOAA was used in this study. AOAA administration (10 mg/kg body weight, for consecutive 5 days) significantly reduced the expression of Na_V_1.7 and Na_V_1.8 when compared with NS injection (Fig. 7B, AOAA/NS = 0.57; n = 3 for each group; *p<0.05, two sample *t*-Test. Fig. 7D, AOAA/NS = 0.46; n = 3 for each group; *p<0.05, two sample *t*-Test). Thus, AOAA treatment reverses the upregulation of Na_V_1.7 and Na_V_1.8 expression in colon DRGs isolated from HIS rats.

## Discussion

In this study we have showed for the first time that heterotypic intermittent stress induced an upregulation of the endogenous H_2_S-producing enzyme CBS expression in colon related DRGs, which was associated with visceral hypersensitivity of rats in responses to colorectal distention. Furthermore, CBS inhibitor significantly reduced the AWR scores and increased the distention threshold, suggesting that endogenous H_2_S-producing enzyme CBS plays an important role in the chronic stress induced visceral hypersensitivity.

CBS and CSE are two important enzymes for generation of endogenous H_2_S in mammals [11], [12], [33], [34]. They have been found in many types of mammalian cells including the central nervous system and peripheral tissues as well [20], [35]. Previous studies have shown that CBS, but not CSE, is expressed by colon-specific sensory neurons, where it is localized to nociceptive neurons [14]. The present studies further demonstrated that CBS was upregulated in a rat model of visceral hyperalgesia induced by chronic intermittent stress, indicating that CBS might be a major enzyme responsible for the endogenous production of H_2_S in these cells under chronic stressed conditions. However, expression of CSE was not altered in this rodent model of visceral pain (Fig. 1D), suggesting that CSE may not be a major factor involved in the visceral pain induced by chronic stress. Further experiments are needed to determine whether the activity of CSE such as phosphorylation was enhanced in this model. Of note is that the time course of CBS upregulation was not completely paralleled with the time course of the enhanced AWR scores. AWR scores only lasted for 24 hours and returned to baseline 48 hours after termination of the last stressor (Figure 1A and B) while the CBS expression lasted for 48 hours (Figure 1C). The detailed reason for this inconsistency remains unknown but the trend in reduced expression of CBS and/or an increase in endogenous antinociceptive substances such as opioids would be the major contributors after chronic stress [36].

Although the detailed mechanism for upregulation of CBS expression is unknown, our data provides additional evidence to confirm the idea that CBS plays an important role in stress-induced visceral hypersensitivity. AOAA, an inhibitor of CBS, significantly mitigates visceral hyperalgesia in HIS-treated rats, in a dose-dependent manner (Fig. 2). The reason for using AOAA but not hydroxylamine (HA) in the present study is because HA has a COX-1 inhibitory action [37]. Since AOAA did not produce any significant effect in healthy control rats (Fig. 2D), AOAA-induced effect was not a nonspecific analgesic effect. This also suggested that the role of CBS in signaling colonic distension may not be as important in health as in the sensitized state. Furthermore, AOAA treatment remarkably decreased amplitude of peak sodium current densities of colon-specific DRG neurons (Fig. 3), indicating the reduction in AWR scores by AOAA may be attributed to the reduced sodium currents. DRG neurons express both TTX sensitive and TTX insensitive voltage-gated sodium currents [38], [39]. Although we did not isolate these two currents by patch clamp studies, our gene expression data demonstrated that HIS dramatically upregulated both Na_V_1.7 and Na_V_1.8 expression, which are predominantly expressed by DRG neurons and involved in chronic pain conditions [40], [41]. The enhanced expression of these two subunits of VGSCs contributes to the enhanced current density and thus leads to an enhanced excitability of colon specific DRG neurons. More importantly, we showed here for the fist time that CBS inhibitor reversed the upregulation of both Na_V_1.7 and Na_V_1.8 expression. This finding would well explain why CBS inhibitor reduced the hyperexcitability and mitigated the visceral hyperalgesia in HIS rats. In addition, NaHS, the donor of H_2_S, greatly enhanced the excitability of colon-specific DRG neurons *in vitro* (Fig. 6). This conclusion was supported by our observations that NaHS significantly decreased the rheobase, hyperpolarized the AP threshold and increased the number of APs evoked by 2 times current stimulation. Similarly, HIS also enhanced the neuronal excitability of colon specific DRG neurons, which is in keeping with our previously published data [29]. These data suggest that NaHS mimics the effect of HIS on neuronal excitability, further indicating that CBS-H_2_S signaling was involved in the development of visceral pain induced by heterotypical intermittent stress.

Another important observation we made is that NaHS greatly enhanced the amplitude of peak sodium current densities of colon-specific DRG neurons *in vitro* (Fig. 5). The increase in sodium current density may well contribute to the enhanced excitability of colon specific neurons. Although the detailed mechanisms by which H_2_S induces visceral hyperalgesia have yet to be fully investigated, our data and that of others suggest that colonic nociceptors are a prime site of action. Matsunami et al [25] suggested that intracolonic NaHS might activate or sensitize T-type Ca^2+^ channels, thus produced visceral nociceptive behavior. Maede et al [42] have demonstrated intrathecal administration of NaHS caused significant decrease in mechanical nociceptive threshold in rats, which is mediated by activation or sensitization of T-type Ca^2+^ channels (Ca_V_3.2) expressed in the primary afferents and/or spinal nociceptive neurons. In this study, we have provided new evidence to support the view that H_2_S donor NaHS enhanced excitability of colon specific DRG neurons via sensitization of voltage-gated sodium channels (Fig. 5 and 6). Although application of NaHS mimics the effect of HIS on sodium channel activities (Figure 3C), the underlying mechanism might differ. The acute application of NaHS is unlikely to increase the channel expression, thus led to potentiation of peak sodium current densities. However, HIS significantly enhanced expression of Na_V_1.7 and Na_V_1.8. Together, these results suggest that H_2_S-induced hyperalgesia and pro-nociception seems to be related to the sensitization of T-type Ca^2+^ channels, TRPV1 channels, TRPA1 channels [43] and Na_V_ channels depending on different environmental stimuli. It is of note that H_2_S is reported to relax colonic smooth muscles via opening of ATP-sensitive K^+^ channels [17], [21] or to activate μ opioid receptors [23], thus producing anti-nociceptive effect. This discrepancy might be arisen from H_2_S concentration, effect of inflammation on H_2_S action and H_2_S action sites. Further researches are needed to determine the roles of H_2_S in health and disease.

In conclusion, we have demonstrated that inhibition of CBS-H_2_S signaling pathways significantly mitigates visceral hypersensitivity induced by heterotypical intermittent stress. In particular, CBS inhibitor suppresses voltage-gated sodium channel currents of colon specific DRG neurons and reverses the enhanced expression of Na_V_1.7 and Na_V_1.8 subtypes. These findings emphasize a crucial role for endogenous hydrogen sulfide producing enzyme CBS in visceral hyperalgesia, thus identifying a potential target for novel agents for the treatment of visceral pain in IBS and related disorders.
